# Influence of omega-6 PUFA arachidonic acid and bone marrow adipocytes on metastatic spread from prostate cancer

**DOI:** 10.1038/sj.bjc.6605481

**Published:** 2009-12-08

**Authors:** M D Brown, C Hart, E Gazi, P Gardner, N Lockyer, N Clarke

**Affiliations:** 1 Genito-Urinary Cancer Research Group, School of Cancer, Enabling Sciences and Technology, Paterson Institute for Cancer Research, The University of Manchester, Manchester Academic Health Science Centre, The Christie NHS Foundation Trust, Manchester M20 4BX, UK; 2 School of Chemical Engineering and Analytical Sciences, Manchester Interdisciplinary Biocentre (MIB), The University of Manchester, 131 Princess Street, Manchester M1 7DN, UK; 3 Department of Urology, The Christie NHS Foundation Trust, Wilmslow Road, Manchester M20 4BX, UK; 4 Department of Urology, Salford Royal Hope NHS Foundation Trust, Stott Lane, Salford M6 8HD, UK

**Keywords:** arachidonic acid, bone marrow, prostate cancer, lipid, metastasis, adipocytes

## Abstract

**Background:**

Prostate cancer (CaP) preferentially metastasises to the bone, and we have previously shown that the poly-unsaturated fatty acid (PUFA) arachidonic acid (AA) is a potent stimulator of CaP invasion. Here we present that AA promotes CaP invasion by inducing bone marrow adipocyte formation.

**Methods:**

Boyden invasion-chamber assays assessed the ability of dietary oils, their PUFA components, and specific PUFA-loaded adipocytes to induce PC-3 invasion. Lipid transfer and metabolism was followed using deuterated AA and Fourier Transform Infrared spectroscopy (FTIR).

**Results:**

Poly-unsaturated fatty acid constituents, but not their corresponding dietary oils, induced PC-3 invasion. PUFAs induce bone marrow adipocyte (BM-Ad) differentiation with AA inducing higher levels of BM-Ad differentiation, as compared with other PUFAs (3998±514.4 *vs* 932±265.8; *P*=0.00002), which stimulated greater PC-3 invasion than free AA (22 408.5±607.4 *vs* 16 236±313.9; *P*=0.01111) or adipocytes generated in the presence of other PUFAs. In bone marrow co-culture PC-3 and BM-Ad interactions result in direct uptake and metabolism of AA by PC-3 cells, destruction of the adipocyte and subsequent formation of a bone metastasis.

**Conclusion:**

The data supports the hypothesis that AA not only promotes CaP invasion, it also prepares the ‘soil’, making it more supportive for implantation and propagation of the migrating metastatic cell.

A high proportion of young men have microscopic evidence of prostate cancer (CaP) with prevalence increasing with age (Franks, 1954). Notwithstanding the global similarity in prevalence, the incidence of clinical CaP/CaP death varies widely internationally. In western societies, clinical CaP is common compared with developed non-western countries, for example Japan (Ferlay *et al*, 2004). Migrational studies also show that movement from low- to high-risk countries raises CaP risk in migrants to that in native residents, (Crawford, 2003) suggesting that environmental factors affect CaP's clinical incidence and mortality.

Epidemiological studies highlight differences in the omega-6 (*ω*-6) to omega-3 (*ω*-3) polyunsaturated fatty acid (PUFA) ratio between regions of high and low CaP risk. PUFA makes up 19–22% of fat energy intake in the United States of America, with 84–89% coming from the *ω*-6 linoleic acid (LA). The *ω*-3 *α*-linolenic acid (ALA) only contributes 9–11% of PUFA-related energy (Kris-Etherton *et al*, 2000), giving an *ω*-6 : *ω*-3 ratio of 10.6 : 1. This compares with 4 : 1 in Japan and is well above the perceived optimal ratio of 2.3 : 1 (Sugano and Hirahara, 2000). It is interesting to note that the increases in incidence and mortality from CaP in developing countries over the last 30 years has coincidentally mirrored the increased dietary *ω*-6 : *ω*-3 PUFA ratio brought about by greater vegetable oil consumption and the use of cereal grain to feed livestock.

Diets enriched with high levels of *ω*-6 PUFAs, particularly LA and its metabolite arachidonic acid (AA), are associated with poorer CaP prognosis (Wynder *et al*, 1994; Rose, 1997). The precise mechanism of this is unclear. Arachidonic acid exerts influence through its COX-2 and LOX metabolites, inducing malignant CaP proliferation (Hughes-Fulford *et al*, 2006), inhibiting apoptosis (Ghosh, 2004), inducing angiogenesis (Nie *et al*, 1998), and inducing disease progression (Honn *et al*, 1994; Norrish *et al*, 1999). Studies of the 5-LOX product, 5-HETE, show this is as a key regulator of tumour aggressiveness. 5-HETE protects prostate cells from apoptosis (Ghosh, 2004) and is crucial in EGF-related cellular proliferation (Hassan and Carraway, 2006). Arachidonic acid itself is a potent stimulator of malignant prostate epithelial cell (PEC) invasion, and is able to recover invasion towards adipocyte-free human bone marrow stroma (BMS) by the AA metabolite PGE_2_. This stimulatory effect is abrogated by the addition of long chain marine *ω*-3 PUFAs eicosapentaenoic acid (EPA) and docosahexaenoic acid (DHA) (Brown *et al*, 2006). It has also been hypothesised that AA acts as a secondary messenger involving an autocrine loop-maintaining EGFR activation (Angelucci *et al*, 2008).

Prostate cancer metastasises preferentially to the BMS. The reasons underlying this are uncertain but evidence suggests that BM adipocytes (BM-Ad) are of importance. The precise role of BM-Ad, present in abundance in BMS, is unknown. It is hypothesised that they have a role in haematopoiesis (Gimble *et al*, 1992) or act as energy stores supporting oxidative metabolism of resorbing osteoclasts (Dodds *et al*, 1994) and are fundamental to BMS formation and its long-term maintenance (Gartner and Kaplan, 1980). In long-term human BMS/CaP models malignant cells migrate towards BM-Ad and take up lipids from adipocytes or the surrounding microenvironment (Brown *et al*, 2006). Separate studies by Tokuda *et al* (2003) and Gazi *et al* (2007) found that PC-3 cells interacted directly with adipocytes and using Fourier Transform Infrared spectroscopy (FTIR) to follow deuterated palmitic acid, Gazi *et al* (2007) confirmed the direct uptake of lipid by CaP cells from BM-Ad.

Polyunsaturated fatty acids acting *in vitro* are potent inducers of adipocyte differentiation (Madsen *et al*, 2005), but the roles of dietary PUFAs and their effect on BM adipogenesis and CaP metastasis are unknown. Here we show that dietary PUFAs themselves are not strong stimulators of CaP invasion but require adipocyte processing. We also show that AA induces invasion itself and induces differentiation of BM mesenchymal stem cells (MSC) into adipocytes, which are themselves potent inducers of invasion. Together the data suggests that a high level of AA derived from dietary intake is a major risk factor for CaP progression.

## Materials and methods

### Materials

All general reagents were purchased from Sigma-Aldrich (Poole, UK), including all the lipids except AA (MP Biomedicals, London, UK). All lipids except D_8_-AA were either made up in ethanol or methyl-*β*-cyclodextrin to produce a 10 mg ml^−1^ emulsion. D_8_-AA was supplied in methyl acetate, which was removed by evaporation under a nitrogen gas stream at room temperature before dissolving in ethanol. All tissue culture reagents were from Invitrogen (Paisley, UK) except Hygromycin B, which was obtained from PAA Laboratories (Yeovil, UK). Foetal calf serum (FCS) was supplied by Labtech International (Uckfield, East Sussex, UK). Matrigel basement membrane matrix and 8 *μ*m FluoroBlok cell culture inserts were from BD Biosciences (Oxford, UK). RosetteSep MSC enrichment cocktail and MesenCult basal media were supplied by StemCell Technologies (London, UK).

### Cell lines and primary BMS culture

PC-3 (ATCC, Manassas, VA, USA) and the PC3-GFP cell lines were cultured in Ham's F12, 7% FCS and 2 mM L-glutamine, with the addition of Hygromycin B (0.15 mg ml^−1^) for the GFP variant, at 37°C, 5% CO_2_ in air. BMS was cultured from human ribs removed for access during routine renal surgery for non-malignant disease after informed consent. BMS cultures were prepared according to the method of Coutinho *et al* (1993) and cultured in long-term culture medium (Iscove's Modified Dulbecco's Medium containing 10% FCS, 10% horse serum and 50 *μ*M hydrocortisone) or used for MSC isolation.

### Mesenchymal separation from normal rib

Mesenchymal stem cells were isolated from human donor red cell-enriched rib bone marrow using the RosetteSep human MSC enrichment cocktail and cultured as defined by Gazi *et al* (2007). Briefly, human donor red cell-enriched rib bone marrow was incubated with RosetteSep antibody cocktail (50 *μ*g ml^−1^) for 20 min at room temperature. Cells were diluted with two volumes of PBS+2% FCS, 1 mM EDTA before centrifuging at 300 **g** for 25 min at room temperature over a Ficoll-Paque cushion. Enriched cells were plated at 1 × 10^7^ cells 25 cm^−2^ flask in complete media (90 ml MSC basal medium/10 ml MSC stimulatory supplement) and incubated at 37°C, 5% CO_2_ in air. After 24 h, non-adherent cells were removed and fresh complete media added. Media was refreshed every 3–4 days until confluent. Adipogenesis was induced by replacing the basal media with proprietary MSC adipogenic media or with MSC basal medium containing 50 *μ*M hydrocortisone and 50 *μ*M lipid. Cultures were incubated at 37°C, 5% C0_2_ in air and media was refreshed every 14 days until adipocytes were present.

### Invasion assay

A variation of the invasion co-cultures described by Hart *et al* (2005) was used. Briefly, FluoroBlok cell culture inserts (8 *μ*m) coated with Matrigel diluted 1 : 25 with phenol red free RPMI 1640 medium, were placed in a 24-well plate containing 1 ml of RPMI 1640 (w/o phenol red)/0.1% fatty acid free (FAF) BSA/10 mM HEPES with either tissue culture plastic (TCP), BMS, mBM-Ad or lipids in the base. PC-3 GFP cells (2 × 10^5^ cells in 0.25 ml of RPMI 1640 /0.1% FAF BSA) were seeded on top of the inserts. Following incubation at 37°C for 24 h invasion was assessed on a BMG FLUOstar OPTIMA plate reader at 488/520 nm (excitation/emission filter). The number of adipocytes in each well was scored post fixation with 4% Formalin for 20 min at room temperature.

### Cell fixation

D_8_-arachidonic acid labelled adipocytes on MirrIR plates were washed twice with PBS and seeded with 1 × 10^5^ 24 h serum-starved PC-3 cells. After 48 h of incubation in serum-free RPMI at 37°C, 5% CO_2_ in air cultures were washed twice with PBS and fixed in 4% paraformaldehyde for 25 min. Cells were washed thrice for 5 min each in Sorensen's buffer (0.15M, pH 7.4) and post-fixed in 1% OsO_4_ for 1 h. Cells were washed thrice for 5 min each with Sorensen's buffer before dehydration using increasing concentrations of ethanol : water. Cells were then dried using a critical point drier. The ethanol was substituted for liquid CO_2_ following six ethanol–CO_2_ exchanges and a 5 min immersion (repeated nine times). Phase-transition was induced by heating the chamber to ∼45°C at 1200 psi. Preservation was assessed by microscopy. The cells were stored in a desiccator until FTIR analysis.

### FTIR micro-spectroscopy

High-definition FTIR micro-spectroscopic maps of paraformaldehyde–OsO_4_–CPD fixed adipocyte–PC-3 cell co-cultures were collected in rapid-scan reflectance mode at 6.25 *μ*m pixel resolution using a Perkin Elmer Spotlight spectrometer with a 16 × 1 MCT linear-array detector. The background scan was recorded at 8 or 4 cm^−1^ spectral resolution with 75 scans, whereas the sample scan was recorded at 8 or 4 cm^−1^ spectral resolution with 64 scans.

### Time-lapse microspectroscopy

Confluent adipogenically active human BMS in T12.5 flasks were seeded with serum starved PC-3 cells and observed using a time-lapse video microscope capturing one frame every 20 s (Allen, 1987).

### Statistics

All values are presented as mean±s.e.m. All assays were compared with use of the two-tailed Student's *t*-test. A threshold of significance was set at *P*<0.05.

## Results

### Dietary oils do not induce invasion, but their PUFA components do

In order to determine the ability of common dietary oils used in western diets, which contain different levels of *ω*-3/6/9 lipids, along with pure individual PUFAs to stimulate CaP migration we encapsulated the lipids in methyl-*β*-cyclodextrin to create an emulsion suitable for cell culture.

**Figure 1:**
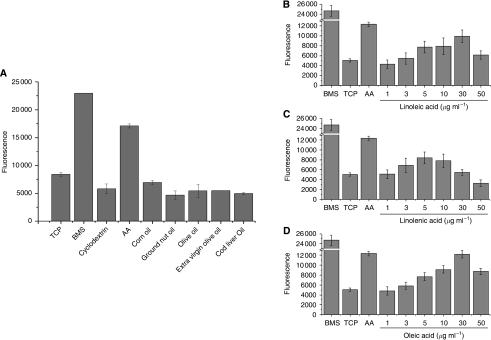
Poly-unsaturated fatty acid (PUFA) components, but not heterogeneous dietary oils, are stimulators of prostate cancer (CaP) invasion. 2 × 10^5^ PC-3-GFP cells were seeded in modified Boyden chambers above either (**A**) 1 *μ*g ml^−1^ dietary lipids or increasing levels of (**B**) linoleic acid (LA) (**C**) *α*-linolenic acid (ALA) (**D**) oleic acid encaged in methyl-*β*-cyclodextrin. Levels of invasion are proportional to fluorescence detected by a bottom reading BMG FLUOstar OPTIMA plate reader at 488/520 nm (excitation/emission filter). In each assay (*n*=3) a serum free RPMI 1640 (TCP)^–ve^ control, human BMS^+ve^ control and a 3 *μ*g ml^−1^ arachidonic acid (AA) control were included.

Bone marrow stroma control and AA, previously shown to induce maximum PC-3 invasion (Brown *et al*, 2006), induced significant invasion through Matrigel (Figure 1A). Common dietary oils failed to induce significant invasion compared with controls. Analysis of specific PUFAs in isolation showed induction of PC-3 invasion. Figure 1B-D shows increased invasion induced by increasing concentrations of LA, ALA and oleic acid. The AA precursor LA at a maximum concentration of 30 *μ*g ml^−1^ induced invasion to a level approaching 3 *μ*g ml^−1^ (10 *μ*M) AA (9925.7±1253.1 *vs* 12 347.2±455; *P*=0.09935). Both ALA (*ω*-3) and oleic acid (*ω*-9) induced PC-3 invasion (8406±1202.9 *vs* 5054±334.7; *P*=0.02291: LA and TCP control, respectively), but at a significantly lower rate than that from 3 *μ*g ml^−1^ AA (*P*=0.01195). The invasive stimulus closest to AA came from 30 *μ*g ml^−1^ oleic acid, which induced equivalent levels to AA (12 192.8±790.6 *vs* 12 347.2±455; *P*=0.00001).

### PUFAs stimulate adipocyte differentiation in BMS

Prostate cancer cells were not stimulated to invade by complex lipid mixtures in common dietary oils (Figure 1), but were stimulated when PUFAs were presented on their own. Lipids are processed and stored within adipocytes and CaP preferentially metastasise to the adipocyte-rich red BM. Therefore, we sought to determine the effect of PUFAs on adipocyte formation in BM and on the adipocytes ability to induce CaP invasion.

**Figure 2:**
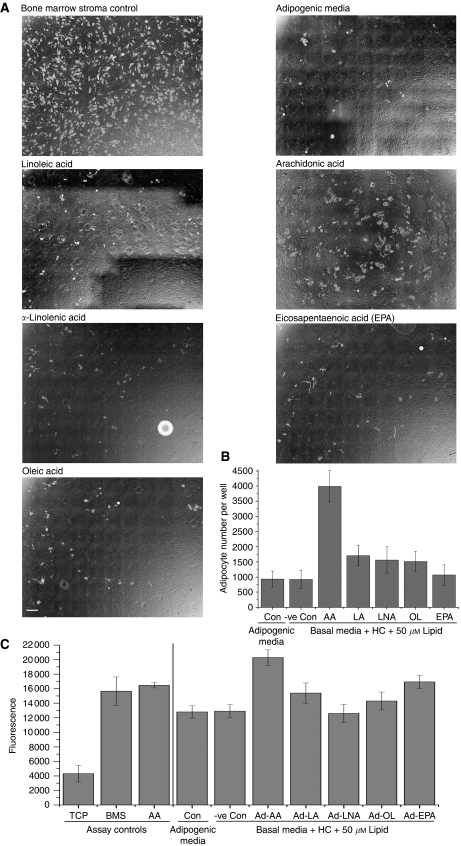
Arachidonic acid (AA) primes the ‘soil’ for prostate cancer (CaP) invasion. Mesenchymal cells isolated from primary human bone marrow (BM) were grown in the presence of 50 *μ*M linoleic acid (LA), arachidonic acid (AA), *α*-linolenic acid (ALA), eicosapentaenoic acid (EPA) or oleic acid in basal media supplemented with 5 × 10^−7^ M hydrocortisone to induce adipocyte differentiation. Control cultures include differentiation in proprietary adipogenic media and long-term bone marrow stroma (BMS) growth media. (**A**) Phase contrast photo-micrographic mosaics comprising 11 × 8 overlapping fields of view merged using the Mosaic J plug-in (ImageJ). Adipocytes are distinguished from dark stromal background as phase-bright cells. Scale bar=1 mm. (**B**) Histogram showing number of adipocytes formed in the presence of each lipid (*n*=3). (**C**) Histogram showing the invasive stimulus of specific lipid-laden adipocytes derived from human BM mesenchymal stem cells (MSC) with 5 × 10^−7^ M hydrocortisone. 2 × 10^5^ PC-3-GFP cells were seeded in modified Boyden chambers and invasion was measured after 18 h using a bottom reading BMG FLUOstar OPTIMA plate reader at 488/520 nm (excitation/emission filter).

Bone marrow MSCs isolated from human ribs were differentiated down the adipocyte lineage (mBM-Ad) by culture in mesenchymal stem cell basal media containing hydrocortisone and specific PUFAs. Control MSCs were cultured in proprietary adipogenic media (StemCell Technologies), containing an undisclosed lipid mixture and long-term BMS culture from the same patient. All PUFAs, except AA, induced generation of similar adipocyte numbers within each culture compared with commercial adipogenic media and hydrocortisone ‘lipid free’ negative control. Arachidonic acid however, induced markedly greater numbers of adipocytes than adipogenic media (3998±514.4 *vs* 932.3±265.8, respectively; *P*=0.00002) (Figure 2).

### PUFA-loaded adipocytes, especially AA, induce invasion

The ability of specific lipid pulsed mBM-Ad to stimulate CaP invasion through Matrigel was compared with invasive stimulation by BMS grown under standard conditions and mBM-Ad derived using adipogenic media (Figure 2C). Adipocytes generated by adipogenic media stimulated PC-3 invasion although this was significantly less than that induced by 3 *μ*g ml^−1^ AA (11 469±1151.53 *vs* 16 236±313.9, respectively; *P*=0.00409). It is interesting to note that the level of invasion induced by the adipogenic media was similar to that induced by mesenchymal cells differentiated by hydrocortisone without exogenous lipids (11 165.8±871 *vs* 11 469±1151.53; *P*=0.93959). This phenomenon was observed with adipocytes differentiated in the presence of ALA, which stimulated PC-3 invasion at similar levels to controls (12 429.25±1002.847 *vs* 11 165.8±871 (*P*=0.89279) and 1469±1151.53 (*P*=0.65767) for adipogenic media and no lipid control, respectively) and was significantly different to the free AA positive control (*P*=0.0241).

Adipocytes loaded with *ω*-6 LA or with *ω*-9 oleic acid both induced invasion at similar levels to 3 *μ*g ml^−1^ free AA (16 459±1261.6 and 14 596±696.19 *vs* 16 236±313.9; *P*=0.52990 and *P*=0.16877). Invasion was greater than that induced by adipogenic media and the ‘no lipid’ controls, but the increase did not reach significance (11469±1151.5 *P*=0.13418 and 0.05861, 11165.8±871 *P*=0.33484 and 0.14815 respectively). Surprisingly, EPA-dosed adipocytes stimulated a significantly greater invasive stimulus than either negative control (*P*=0.00543 and 0.0023) achieving levels similar to 3 *μ*g ml^−1^ free AA (16 927.6±928.7 *vs* 16 236±313.86 *P*=0.6887).

The most striking effect was seen in adipocytes formed in the presence of AA, these generated the greatest invasive stimulus for PC-3 cells compared with negative lipid free control (22 408.5±607.4 *vs* 11 165.8±871 *P*=0.00005). The stimulus was greater than that induced by free AA within the media of the bottom chamber (16 236±313.9 *P*=0.01111 BM-Ad–AA *vs* free AA, respectively).

### CaP cells within BMS migrate to and interact with adipocytes

mBM-Ad derived from BM mesenchymal cells grown with AA are potent stimulators of PC-3 invasion. Whether this is because of leeching of PUFAs into the surrounding media from adipocytes, producing higher local concentrations or to undetermined adipocyte-related factors is unclear.

**Figure 3:**
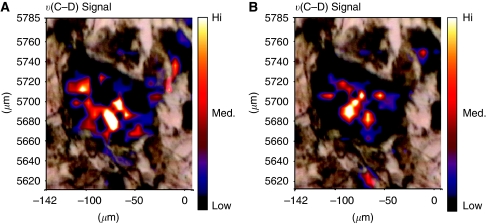
Localisation of D_8_-AA within bone marrow (BM) adipocytes. Deuterated signal overlaid onto phase contrast photomicrograph of a D_8_-AA loaded BM adipocyte. BM mesenchymal stem cells (MSC) were differentiated into adipocytes with 50 *μ*M D_8_-AA. Phase contrast image of an adipocyte overlaid with (**A**) *υ*(C–D) Fourier Transform Infrared spectroscopy (FTIR) spectral image and (**B**) *υ*(=C–D) spectral image to show localisation of D_8_-AA.

As migrating CaP cells within the BMS take up lipids (Brown *et al*, 2006) we utilised mBM-Ad pulsed with D_8_-AA to determine whether these cancer cells take up lipids released from the mBM-Ad into the BMS environment or whether the process is mediated through direct interaction with the adipocytes themselves. A white light micrograph (Figure 3) shows a mBM-Ad overlaid with the FTIR spectral maps correlating to the vibrational spectral characteristics *υ*(C–D) and *υ*(=C–D) of D_8_-AA and demonstrates clearly that D_8_-AA lies within the adipocyte.

**Figure 4:**
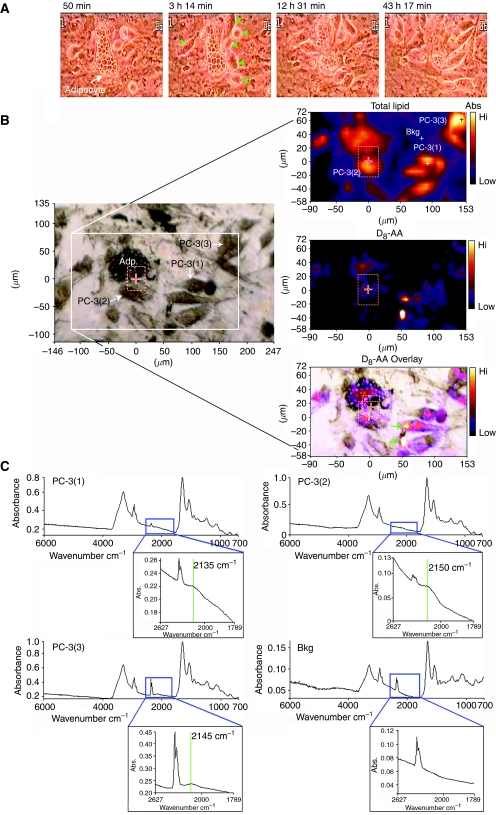
PC-3 cells target and take-up arachidonic acid (AA) from AA-loaded human bone marrow adipocyte (BM-Ad). (**A**) Interaction of PC-3 cells co-cultured with AA-pulsed adipocytes from human BM mesenchymal cells followed by time-lapse video-microscopy. Adipocyte=white arrow, PC-3 cells=green arrows. (**B**) Photomicrographs showing separate phase contrast, total lipid and D8-AA Fourier Transform Infrared spectroscopy (FTIR) spectral image and a phase contrast D_8_-AA overlaid image of a PC-3 D_8_-AA loaded BM adipocyte (Adp) co-culture. (**C**) Fourier Transform Infrared Spectroscopy spectra of four regions defined in the phase contrast and total lipid spectra photomicrograph shown in B, with inset high-resolution spectra of the 1789–2627 cm^−1^ region corresponding to D_8_-AA. Bkg=background.

Using time-lapse video-microscopy we followed the migration of serum-starved PC-3 cells in co-culture with BMS containing D_8_-AA-loaded adipocytes (Figure 4A and video, Supplementary Figure 3). Binding of the rounded PC-3 cells to BMS was complete within 120 min following addition of the cells. The PC-3 cells migrated towards adipocytes with first contact observed after approximately 2.5 h. Over the next 2 h the PC-3 cells appeared to flatten, taking on a mesenchymal morphology. During this period the size of the upper adipocyte in the viewing field decreased in size as it was destroyed, and that the lipid vesicles within adipocytes in the lower aspects of the field began to alter, becoming smaller. The destruction of the upper adipocytes continued over the next 46 h, with adipocytes being disrupted after approximately 12.5 h and with almost complete loss of the adipocyte/intra-adipocyte lipid vesicles at 48 h. The lower adipocyte was still present at the end of the co-culture, but it had undergone marked changes in its morphology, intra-cellular lipid vesicles, which became smaller in size, along with a reduction in overall cell size. During this process there was a synchronous and obvious visible increase in the number of CaP cells recruited to adipocytes, with clear morphological change to a mesenchymal phenotype and a marked increase in CaP cellular motility.

### Uptake of D_8_-AA from BM-Ad

Time-lapse video-microscopy data shows CaP cells interact with adipocytes resulting in loss of lipids from the adipocytes. However, time-lapse does not provide information on mechanisms of AA uptake within this environment. Lipid could be taken up by direct interaction between adipocytes and CaP cells or by assimilation from the surrounding media once AA is released by adipocytes following cellular interaction or after the rupture of lipid micelles during the destructive process. AA uptake can be followed by time-lapse microscopy and FACS using Nile Red staining (Brown *et al*, 2006). However, this approach does not distinguish whether AA comes directly from adipocytes, from free AA, or from AA derived from other lipid sources. Utilising D_8_-AA and FTIR spectroscopy, the transit of AA from adipocytes to the CaP cells can be traced (Figure 4).

Figure 4B shows an optical photomicrograph of PC-3 cells below and to the right of an adipocyte. FTIR molecular images obtained from the boxed area and processed to display intensity distribution of the total lipid hydrocarbon and υ(C–D) signals, indicate the location of D_8_-AA and/or its deuterated metabolites. The final panel overlays the υ(C–D) signal on the optical photomicrograph of the region providing spatial localisation of the D_8_-AA. This enables viewing of each cell within the imaging field and acquisition of spectra from site-specific locations, CaP cells and the surrounding stromal matrix to determine D_8_-AA uptake. The total lipid spectra provide the molecular location of each cell within the field of chemical analysis. Analysis of D_8_-AA, shown by the υ(C–D) signal around 2150 cm^−1^, shows that D_8_-AA is located at high levels within the adipocyte and in CaP cells. Background spectra were obtained from the location denoted ‘Bkg’, which show that the peak within the υ(C–D) spectral range is absent, showing that the D_8_–AA and/or its metabolites are contained within the cellular fraction, specifically within adipocytes and CaP cells, but not within stromal cells.

**Figure 5:**
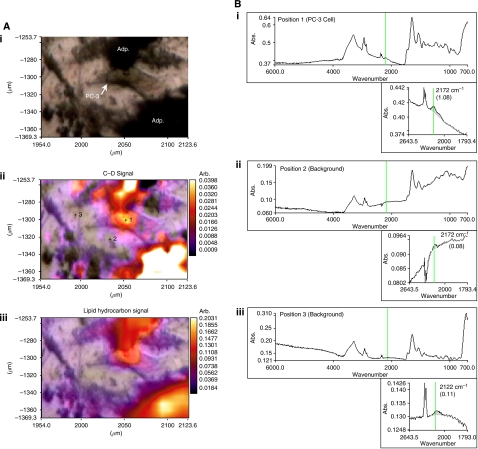
Arachidonic acid (AA) localises around the nucleus following uptake by PC-3 cells. (**Ai**) Phase contrast image of a PC-3/bone marrow adipocyte (BM-Ad) co-culture showing a PC-3 cell flanked top and bottom by D_8_-AA loaded adipocytes; (**Aii**) Fourier Transform Infrared spectroscopy (FTIR)-spectral micrograph of the *υ*(C–D) signal overlaid on to the optical photomicrograph, with increasing signal absorbance depicted by a shift towards red/white colours; (**Aiii**) FTIR-spectral micrograph of the total lipid hydrocarbon signal overlaid onto the optical photomicrograph with increasing signal absorbance depicted by a shift towards red/white colours. (**B**) Fourier Transform Infrared spectroscopy-spectral signatures from i) PC-3 cell, (ii+iii) background as defined in Figure 6Ai and Aii. Fourier Transform Infrared spectroscopy Spectral profiles (inset) concentrate on the 1793.4–2643.5 cm^−1^ region encompassing the (**C**, **D**) spectral region (highlighted by the green vertical line).

Intra-cellular localisation of D_8_-AA and/or its metabolites was determined using greater magnification of the infrared spectral image. Figure 5 shows an optical photomicrograph of a PC-3 cell flanked by D_8_-AA loaded mBM-Ad. υ(C–D) spectral maps show that D_8_-AA localises around the PC-3 nucleus, unlike the total lipid–hydrocarbon signal, which is more homogenous throughout the PC-3 cell.

### Temporal effects of D_8_-AA exposure to endogenous biomolecules in PC-3 cells

The intra-cellular location of AA following uptake by CaP cells (Brown *et al*, 2006; Gazi *et al*, 2009) suggests that AA is being used for specific purposes within the cell. Utilising D_8_-AA and FTIR, it is possible to follow spectral changes in the cell's biochemical constituents over time and relate this information to the D_8_-AA signal.

**Figure 6:**
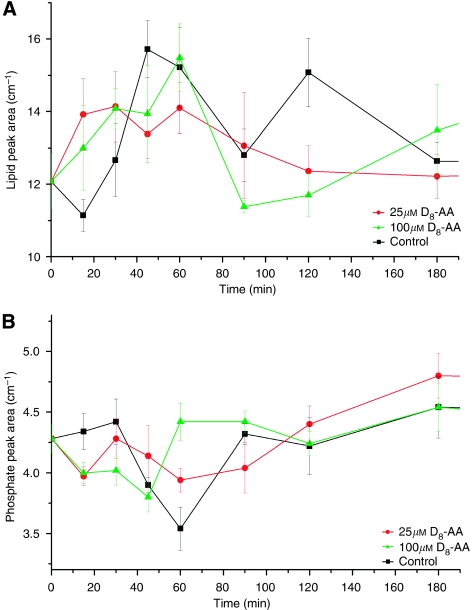
Chemometric analysis of arachidonic acid (AA) metabolism in PC-3 cells. Endogenous (**A**) lipid and (**B**) phosphorylation signals in PC-3 cells following exposure to 25 *μ*M and 100 *μ*M D_8_-AA or no D_8_-AA (control). Fourier Transform Infrared spectroscopy (FTIR) measurements over 3 h.

PC-3 cells in control and 100 *μ*M D_8_-AA conditioned media (Figure 6A) demonstrate an initial rise (0–60 min; *P*= >0.05) in endogenous lipid signal followed by a significant fall. This coincides with an increase in phosphate signal within D_8_-AA exposed CaP cells. A much smaller rise in endogenous lipid signal was observed with 25 *μ*M D_8_-AA at earlier time points. The phosphate intensity for 25 *μ*M D_8_-AA exposed cells did not show significant differences between phosphate intensities at 0 and 90 min (*P*=0.35), but thereafter a rise in mean υ(=C–D) signal was seen, with a significant difference in phosphate signal between 90 and 180 mins (*P*=0.05). This coincided with the decreased mean υ(=C–D) signal observed in the time-course D_8_-AA uptake (Supplementary Figure 2).

## Discussion

The role of lipids in the development and progression of CaP is controversial. The microscopic prevalence of CaP is widespread (Franks, 1954), but the incidence of clinically relevant disease varies internationally, being highest in developed western countries (Ferlay *et al*, 2004). These populations have large numbers of clinically obese men (WHO, 2006) and their general diet is enriched with foods containing high levels of fat. However, the relationship between obesity and incidence of CaP is uncertain (Renehan *et al*, 2008).

Lanier *et al*, 1996 and Rose, 1997 followed changes in Inuit and Japanese population diets, showing that both populations coincidentally experienced increases in clinically significant CaP, as their diet changed from a fish-based diet rich in long chain *ω*-3 lipids (EPA/DHA) to an *ω*-6 enriched westernised diet. It is hypothesized that changes in *ω*-6 : *ω*-3 ratio has led to increased incidence of clinically significant prostate, breast, pancreatic, and colon cancers. This observation mirrors changes in *ω*-6 : *ω*-3 ratio before and after the industrial revolution in ‘western’ countries. Historically, ‘western’ diets had lower saturated and unsaturated fat levels, with *ω*-6 : *ω*-3 ratios of about 1 : 1. The augmented use of modern vegetable oils has increased western *ω*-6 PUFA consumption at the expense of *ω*-3 PUFA (Kris-Etherton *et al*, 2000). During this period the presentation of clinically significant CaP has increased (Hsing *et al*, 2000). Although there are a number of potential reasons for this, such as increased longevity and better CaP detection/diagnosis, it is interesting to hypothesise that the increase is directly related to dietary changes.

As epidemiological evidence suggests that the switch to modern vegetable oils has upset the differential balance of *ω*-6 : *ω*-3 PUFAs resulting in increased risk of developing clinically aggressive CaP, we studied the role of PUFAs in CaP metastatic behaviour utilising validated *in vitro* models (Hart *et al*, 2005), supplemented by time-lapse video-microscopy and functional interrogation using FTIR-based spectroscopy. Oils commonly featured in western diets were made up at concentrations similar to the dose of AA previously shown to induce maximal invasion (Brown *et al*, 2006) and within the concentration range reported by Maurin *et al* 2002 as having an effect on human osteoblasts. Using these oils we were unable to induce CaP cellular invasion in our experiments. This is unlikely to be due to the cyclodextrin used to encage lipid droplets, (required for emulsification of the oil for *in vitro* experimentation) as AA control was similarly prepared. This attracted CaP avidly as reported herein and elsewhere (Brown *et al*, 2006). This finding shows that dietary oils in unprocessed forms are not the factor inducing the observed cellular changes presented herein.

Linoleic acid and ALA both promote proliferation and migration of PC-3 cells, therefore we assessed each lipid component separately as a cyclodextrin-induced emulsion to determine their stimulatory effects. Both induced CaP invasion, with 30 *μ*g ml^−1^ LA inducing the greatest level. The reduced invasion towards LA compared with AA may be because of the cells’ requirement to convert LA into AA and to metabolize AA to induce invasion. It has been postulated (Angelucci *et al*, 2008) that proliferation and migration is controlled by an autocrine loop maintaining EGFR signalling which, in turn, is controlled by AA-induced TGF-*α*. This suggests for LA to stimulate invasion, it must be first metabolised to AA, which is then metabolised by 5-LOX and COX-2 to induce EGFR-mediated signalling of invasion. This may be less efficient at stimulating invasion than supplying AA directly.

*α*-Linolenic acid, a short chain *ω*-3 PUFA (C18) from plant oils, induced high levels of invasion, this was not as pronounced with LA but it occurred maximally at lower concentrations, unlike the long chain *ω*-3 PUFAs, EPA (C20), and DHA (C22), which reduce CaP migration towards BMS (Brown *et al*, 2006). This supports evidence showing that ALA increases the risk of CaP progression (Leitzmann *et al*, 2004).

Invasion towards *ω*-9 oleic acid, the main component of olive oil, was also seen. This conflicts with perceived thinking about olive oil, previously thought to be a major dietary component responsible for reducing the risk of clinically significant CaP in Mediterranean men (Simopoulos, 2004; Stamatiou *et al*, 2007). Oleic acid has been shown to activate EGFR signalling in both endothelial (Vacaresse *et al*, 1999) and breast cancer cell lines (Soto-Guzman *et al*, 2008). Angelucci *et al* (2008) has previously reported that AA stimulates the EGFR pathway leading to increased cellular invasion. This suggests that within our *in vitro* model, where oleic acid is the predominant lipid within the system, oleic acid may act in a similar way to AA and induces invasion via the EGFR signalling pathway.

Differences between dietary oils and their individual constituents in inducing invasion may be because of lipid mixture they contain. We have shown previously that 5 *μ*M EPA or DHA inhibits the invasive effects of 10 *μ*M AA on CaP. It is possible that the ratio of *ω*-6 : *ω*-3 PUFAs in oils tested was within the range which nullified the stimulatory signal of *ω*-6. Other lipids within the mixture may have masked the effect of individual PUFAs. Another possible factor is the form in which prostate cells encounter lipid. Lipids are insoluble and are stored in adipocytes within target tissues. It may be that CaP cells presented directly with neat dietary oils are unable to utilise this substrate. Once the oil mixture has been taken up and ‘processed’ by adipocytes, resulting in production and storage of AA and its metabolites PGE_2_ and 5-HETE, CaP cells may utilise specific lipid or metabolites to induce invasion. If this notion was true, the BM-Ad may be of considerable importance in initiating metastatic implantation. Adipocytes are present in all tissues, but CaP metastasis to subcutaneous tissue is unusual, whereas spread to BM is very common. This raises the question of whether adipocytes in all areas function similarly. Adipocytes in different areas may process/store fat differently according to the role that they are supporting and although few studies have been conducted in this area, it has been reported that BM-Ad's have distinct and critical differences compared with extramedullary adipocytes (Bathija *et al*, 1979; Tavassoli, 1984; Gimble *et al*, 1992).

Although the exact function of BM-Ads is unclear (Nuttall *et al*, 1998) they are numerous in this location, where they have a key role in haematopoiesis (Gimble *et al*, 1992), with cobblestone areas close to adipocytes representing active haematopoiesis. Furthermore, propagation of long term BM culture fails when adipocytes are absent, following arrest of differentiation from adipocyte precursors in steroid-free culture (Brown *et al*, 2006). Therefore, as BM-Ads are integrally involved in haematopoietic cell proliferation/propagation, they may also be an important component in CaP–BMS interaction. This may explain the co-localisation of CaP cells with adipocytes and the consequent influence on CaP migration and growth *in vitro* (Tokuda *et al*, 2003; Brown *et al*, 2006; Gazi *et al*, 2007).

Following these observations, we studied the metabolites and/or AA stored within BM-Ad and their surrounding microenvironment utilising an adipocyte differentiation culture system. This protocol amplifies isolated primary human BM-MSC in culture using basal stem cell media (BSCM) with adipogenic stimulatory supplements to induce differentiation down the adipocyte pathway. Although this method gave rise to BMS containing adipocytes, their number was considerably lower than that achieved with established methods using human BM aspirates in long-term culture (Coutinho *et al*, 1993). This may be because of human aspirates already containing greater numbers of adipocyte precursors (unlike MSC systems, where adipocyte precursors must undergo adipogenic differentiation) and/or to the long-term BM culture media containing selected FCS and horse serum, which support adipogenesis/haematopoiesis. To assess the effect of specific PUFAs on BM-MSC adipogenesis, we utilised BSCM supplemented with hydrocortisone (an essential factor for inducing adipogenesis within BMS cultures), which we have shown previously to be capable of deriving BM-Ad containing specific fatty acids (Gazi *et al*, 2007). We now show for the first time that specific lipids have differential effects on adipocyte differentiation in human BMS. Linoleic acid, ALA, EPA, and oleic acid induced differentiation of similar numbers of adipocytes as the proprietary adipogenic supplements but at the same concentration, AA induced four-fold greater adipogenesis than proprietary media. This suggests that AA interacts with adipogenic differentiation pathways, inducing a proportional increase in adipocyte numbers. Thus, in the presence of specific lipids, the number of BM-Ad will increase, potentially augmenting the chemoattractive stimulus of migrating cancer cells known to be in circulation in escalating numbers as cancer load increases (Danila *et al*, 2007) and increasing the statistical chance of ‘proximity’ encounters between migrating cancer cells and BM-Ad. Thus, in a ‘Darwinian’ sense, the ‘fitness’ of the cancer cell to survive and propagate in red BM is enhanced. Specific PUFAs also had differential effects on cancer cell migration (Figure 2C). There was significant induction by all PUFAs but the AA-loaded adipocytes were especially potent stimulators, surpassing that induced by free AA control. Thus, AA not only induces greater adipogenesis than other PUFAs, it's processing by the adipocyte also leads to super-added chemoattractive stimulation. This may be because of the production of metabolites and initiation of additional pathways that are themselves potent stimulators of invasion (Hassan and Carraway, 2006; Angelucci *et al*, 2008).

The strong stimulation of invasion by EPA-loaded adipocytes is paradoxical, running counter to previous data (Brown *et al*, 2006) showing that marine *ω*-3 PUFAs inhibit CaP invasion. The result herein may be because of adipocytes being loaded with EPA in relative isolation to other lipids. Bagga *et al* (2003) showed that the COX-2 metabolite of EPA, PGE3, although not mitogenic itself, could regulate COX-2 expression and induce IL-6 secretion from macrophages. Although similar in action and signalling to PGE_2_, PGE_3_ is not as efficient in inducing COX-2 gene expression. In our system, where EPA is the main lipid within adipocytes, EPA may be metabolized by the high expression of COX-2, generating PGE_3_ and stimulating IL-6 production. Increased serum IL-6 levels are associated with poorer prognosis in CaP (Nakashima *et al*, 2000) and are known to have proliferative properties which induce cancer-like behaviour, including migration of gastric cancer cells (Lin *et al*, 2007), trans-endothelial migration of melanoma (Kitamura *et al*, 1997) and ovarian cell lines NOMI and SKOV (Obata *et al*, 1997).

We showed previously that CaP cells frequently associate with BM-Ads in co-culture, resulting in the appearance of cytoplasmic lipid vesicles. This observation was supported by Tokuda *et al* (2003), who showed direct interactions of CaP cells with epididymal adipocytes, with concomitant uptake of lipid droplets by CaP cells. Time-linked measurements of co-cultures provide a unique insight to the interaction between CaP cells and BM-Ads. Over 44 h, PC-3 cells migrated towards, made contact with, and induced wholesale destruction of adipocytes (Figure 4A and Supplementary video). When contact was established between epithelial cell and adipocyte, there was a marked induction of cellular activity with dramatic and visible prostate cellular recruitment and proliferation, with concomitant changes in morphology and motility. This suggests that BM-Ads are the focal point for malignant epithelial-cell aggregation and proliferation within BMS and that cellular behaviour can be altered dramatically following lipid BM-Ad contact and lipid uptake. Circulating PECs can be detected in BMS of CaP patients even in early stage disease (Ross *et al*, 2005), but few cells survive to form a metastasis (Melchior *et al*, 1997). Our data suggests that direct interactions of such cells with BM-Ad will enhance their capacity to survive and propagate.

Using FTIR to track deuterated AA in loaded adipocytes, the D_8_-AA signal could only be detected in CaP cells associated with those adipocytes and in remnants of the adipocytes themselves, thus suggesting a specific mechanism of uptake and utilisation of AA by CaP cells. This process was not apparent in stromal cells and is clearly not a process of passive diffusion from the surrounding microenvironment as that the deuterated signal, υ(C–D), could not be detected within the surrounding micro-environment. Objective measurement of direct D_8_-AA uptake by CaP cells from adipocytes supports observations using electron and confocal microscopy (Tokuda *et al*, 2003; Brown *et al*, 2006; Gazi *et al*, 2007) showing direct interaction of CaP cells with adipocytes with direct acquisition of lipid droplets. High-resolution FTIR mapping of lipid laden CaP cells (Figure 5) shows that υ(C–D) localises around the CaP nucleus. Apparent nuclear localisation may occur at the thickest region of the cell, with greater amounts of D_8_-AA (or metabolite) for detection. However, the intensity of lipid hydrocarbon signal across each CaP cell is homogenous, indicating a non-significant change in path-length across these cells. The observed phenomenon is, therefore, likely because of translocation of lipid from the cell membrane towards the nucleus, a process previously observed using Nile Red (Brown *et al*, 2006). This migration of captured lipids may be a conserved mechanism, as measurements using synchroton radiation SR-FTIR of CaP cells co-incubated with D_31_-PA loaded adipocytes also showed FA location to the nuclear region (Gazi *et al*, 2009). The reason for this localisation is unknown and future studies are required to determine which organelles the lipids are localising to.

Fourier Transform Infrared spectroscopy analysis enables the generation of chemical maps of cells, with spectral peaks indicating the presence and relative quantities of specific chemical moieties. Combining this with time-lapse microscopy allows temporal analysis of defined chemical groups, providing the investigator with dynamic metabolic data. These techniques used herein with D_8_-AA showed an initial increase in endogenous lipid signal in CaP cells followed by a drop after 60 min. This drop coincided with an increase in the phosphate signal (Figure 6). Various factors may be contributory, levels of unesterified cytosolic AA are tightly controlled by two distinct, coordinated pathways, for exposure to low or high AA concentrations (Monjazeb *et al*, 2006). The predominant ‘high-affinity–low-capacity’ pathway, incorporates low concentrations of intracellular AA into glycerolipids responsible for phospholipid–arachidonate re-modelling. When the intra-cytoplasmic concentrations of unesterified AA overwhelm this pathway, a ‘low-affinity–high-capacity’ pathway incorporates unesterified AA primarily into TAG and diarachidonyl phospholipids (Monjazeb *et al*, 2006). This may also explain the absence of a similar rise in CaP cells incubated with 25 *μ*M D_8_-AA as the metabolic demand resulting from media exchange was partially compensated by D_8_-AA, which does not contribute to the measured endogenous lipid signal. The influx of D_8_-AA may also have been at sufficiently low levels for the cell to sequester PUFAs using only the high-affinity–low-capacity pathway.

Prostate cancer cells exposed to 100 *μ*M D_8_-AA exhibited significant rises in endogenous lipid signal between 0–60 min (*P*<0.05) (Figure 6A). This may be because of phospholipid biosynthesis and/or TAG synthesis to sequester a large D_8_-AA influx, as part of its regulatory mechanism. The lipid signal for the 100 *μ*M D_8_-AA exposed cells fell significantly after 60 mins, suggesting the initiation of TAG breakdown and mobilisation of D_8_-AA. Between 90–120 mins, where lipid levels were low in 100 *μ*M D8-AA exposed cells, we found a reduction in υ(=C–D) signal (Supplementary Figure 2). These data suggest that mobilized AA is subsequently metabolised by the COX/LOX pathway to eicosanoids and exported out of cells. The significant upregulation of phosphate signal at time-points 60 min (compared with 40 mins), for the 100 *μ*M D_8_-AA exposed cells (Figure 6B), could result from effects of eicosanoids binding to cell surface receptors and activating kinase pathways for stimulating cell growth/proliferation.

In summary, we have shown that dietary oils in their native form do not stimulate CaP progression/invasion unlike their pure constituent PUFAs. This suggests that dietary oils must be processed before they becoming stimulants for invasion. AA induces proportionally higher levels of BM-Ad differentiation than other PUFAs and these AA-loaded adipocytes are potent inducers of PEC invasion. Direct monitoring of CaP cells in BMS co-culture shows that they migrate towards and interact directly with AA-loaded adipocytes. This interaction is followed by direct uptake and metabolism of AA by CaP cells, resulting in the destruction of adipocyte and transformation of the cancer cell into a more aggressive and motile phenotype. Taken together, the data supports the hypothesis that AA not only promotes CaP invasion, it also prepares the BMS ‘soil’, making it more supportive for implantation and propagation of the migrating metastatic cell.
