# Supplementary material for: Influence of omega-6 PUFA arachidonic acid and bone marrow adipocytes on metastatic spread from prostate cancer
Source: Br J Cancer. 2009 Dec 8;102(2):403–13. doi: 10.1038/sj.bjc.6605481 (PMC2816655; doi:10.1038/sj.bjc.6605481)
Supplement: Supplementary Data [file 6605481x1.doc]

**Supplementary Data.**

The isotopically labelled analogue of AA, deuterated AA has the hydrogens at positions 6, 7, 9, 10, 12, 13, 15, 16 replaced with deuterium atoms, which do not alter the chemical structure of AA but alter the CH vibrational spectrum of the molecule (Supplementary Figure 1A). The replacement of the hydrogen atoms with deuterium gives rise to a measurable spectral peak ν(=C-D) peak at ~2250cm^-1^. This peak is located in a frequency range that does not overlap with vibrational signals from endogenous biomolecules, which makes it possible to follow υ(=CD) over time. Note the deuterated carbons are retained within both AA and its metabolites.

Supplementary Figure 1B shows a comparison of spectra (within the region of the C-D signals) from two different cell types treated with two different fixation protocols. Previously, we observed that D_8_-AA incorporated into PC-3 cells fixed with formalin gave rise to a single υ(=C-D) peak at ~2250 cm^-1^. However, FTIR spectra obtained from the lipid sack of D_8_-AA loaded adipocytes (preserved with paraformaldehyde (PF), osmium tetroxide (OsO_4_) and critical-point-drying (CPD)) occasionally demonstrate a peak corresponding to the υ(=C-D) signal together with a broader peak that centres around 2160 cm^-1^ and is within the υ(C-D) range (Figure 1Biv). These peaks originate from the reaction or non-reaction of the double bonds contained within D_8_-AA with OsO_4_. Figure 1C demonstrates that OsO_4_ preserves lipids through forming complexes with the double bonds of unsaturated lipids (Scheme 1) or mediating complexation and cross-linking between unsaturated hydrocarbon chains (Scheme 2). Both reaction schemes should retain the deuterium atoms on the carbon chain.

The double bonds of D_8_-AA in PF-OsO_4_-CPD fixed, D_8_-AA loaded adipocytes, will react with OsO_4_ generating C-D peaks that are part of a heterocyclic compound. The broad peak may be due to a C-D out-of-plane deformation, since the same vibrational mode for the C-H bond can give rise to a broad peak in pyrrole. The presence of both cross-linked and isolated D_8_-AA-OsO_4_ complexes will also contribute to the broad peak. The spectrum acquired from the second example of the lipid deposit of a PF-OsO_4_-CPD fixed, D_8_-AA doped adipocyte, shows a single broad peak at 2160 cm^-1^ indicating that majority or all of this deuterated FA had formed OsO_4_ complexes.

**Figure 1A.** Molecular structures of D_8_-AA. **B.** Raw FTIR spectra (4cm^-1^ spectral resolution) in the C-D region of (i) a formalin-fixed PC-3 cell not incubated with any deuterated fatty acids, (ii) a formalin-fixed PC-3 cell incubated with D_8_-AA, (iii) the lipid deposit of a PF- OsO_4_-CPD fixed adipocyte incubated with D_8_-AA, a second example of (iv) the lipid deposit of a PF-OsO_4_-CPD fixed adipocyte incubated with D_8_-AA. The difference in the number of peaks observed in each spectrum for this spectral region arises from the differences in fixation, either formalin or OsO_4_ fixation. **C.** OsO_4_ reaction with unsaturated hydrocarbon chains to form cyclic esters. Scheme I: Reaction with a single unsaturated hydrocarbon chain. Scheme II: Cross-linking reaction of OsO_4_ with adjacent unsaturated hydrocarbon chains.

**Supplementary Figure 2**

**AA is rapidly taken up / processed by CaP**

Using the fluorescent neutral lipid stain Nile Red we have shown previously AA uptake occurs 5min after lipid induction, reaching maximum levels by 45min. Thereafter, the fluorescent signal decreased, suggesting loss of intracellular AA. To follow the progress of AA in CaP cells we utilized AA labelled isotopically with deuterium (D_8_-AA), imaged with FTIR microscopy, which follows specific molecular groups using their vibrational spectra (supplementary data). Unlike previous studies (14), Figure 2 shows an increasing υ(=CD) signal over time, peaking at 90min and retaining the υ(=CD) signal for 24h. A fall in mean υ(=C-D) signal occurred between 90 and 120min at both AA concentrations. This did not reach statistical significance but the rise in υ(=C-D) signal between 120 and 180min to levels similar to the 90min time point in 25μM and 100μM D_8_-AA exposed cells was significant (p=0.03252 and 0.02494 respectively) suggesting this initial drop is genuine. Two-way ANOVA of the 25μM and 100μM D_8_-AA curves shows no significant difference in υ(=C-D) between the two curves (F=0.097, p=0.996) suggesting that the rate of D_8_-AA uptake does not rely on the external lipid concentration.

**
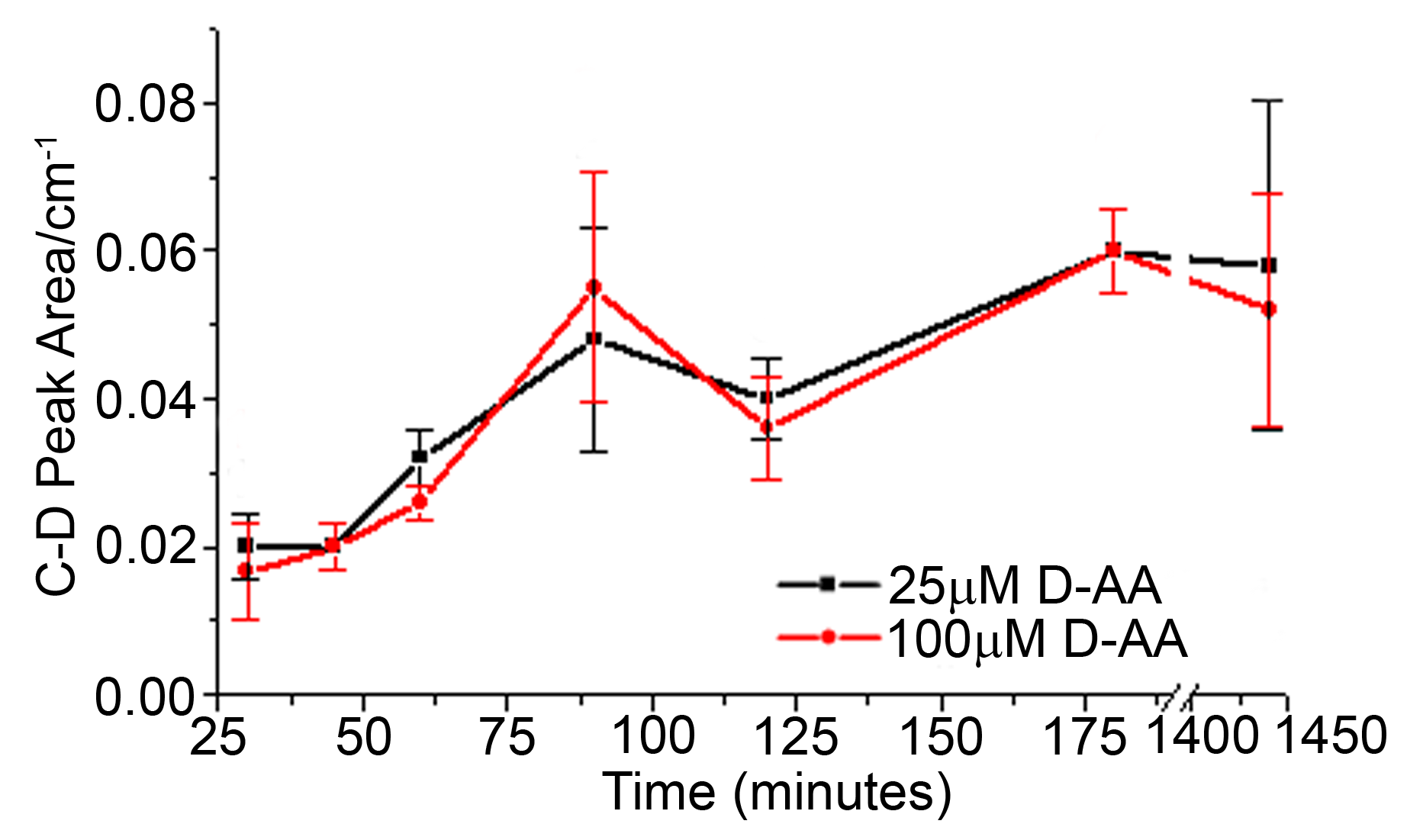
**

**Figure 2:** **PC-3 uptake of D_8_-AA followed by FTIR.** Graph shows the mean intracellular υ(=C-D) signal intensities (±SE) as a function of time in PC-3 cells incubated with 25μM or 100μM D_8_-AA.

**Supplemental Figure 3.**

Time-lapse video-microscopy following the interactions of PC-3 cells co-cultured with AA loaded adipocytes. Human BM mesenchymal stem cells were differentiated in the presence of 50μM AA and 5x10^-7^M hydrocortisone to give rise to BMS containing AA loaded adipocytes. PC-3 cells were added and allowed to bind. A field of view was selected and followed over time using a Leitz Diavert microscope with a 10x phase contrast long working distance objective lens in a heated chamber maintained at 37°C. 1 frame of footage was recorded every 20 seconds using a Dazzle Fusion video capture device and imported into Pinnacle Studio 9 (Pinnacle Systems) for editing.
